# The Alzheimer's Amyloid-Degrading Peptidase, Neprilysin: Can We Control It?

**DOI:** 10.1155/2012/383796

**Published:** 2012-07-26

**Authors:** N. N. Nalivaeva, N. D. Belyaev, I. A. Zhuravin, A. J. Turner

**Affiliations:** ^1^School of Molecular and Cellular Biology, Faculty of Biological Sciences, University of Leeds, Leeds LS2 9JT, UK; ^2^I.M. Sechenov Institute of Evolutionary Physiology and Biochemistry, RAS, 44 Thorez Avenue, Saint Petersburg 194223, Russia

## Abstract

The amyloid cascade hypothesis of Alzheimer's disease (AD) postulates that accumulation in the brain of amyloid **β**-peptide (A**β**) is the primary trigger for neuronal loss specific to this pathology. In healthy brain, A**β** levels are regulated by a dynamic equilibrium between A**β** release from the amyloid precursor protein (APP) and its removal by perivascular drainage or by amyloid-degrading enzymes (ADEs). During the last decade, the ADE family was fast growing, and currently it embraces more than 20 members. There are solid data supporting involvement of each of them in A**β** clearance but a zinc metallopeptidase neprilysin (NEP) is considered as a major ADE. NEP plays an important role in brain function due to its role in terminating neuropeptide signalling and its decrease during ageing or after such pathologies as hypoxia or ischemia contribute significantly to the development of AD pathology. The recently discovered mechanism of epigenetic regulation of NEP by the APP intracellular domain (AICD) opens new avenues for its therapeutic manipulation and raises hope for developing preventive strategies in AD. However, consideration needs to be given to the diverse physiological roles of NEP. This paper critically evaluates general biochemical and physiological functions of NEP and their therapeutic relevance.

## 1. Introduction

The amyloid cascade hypothesis of Alzheimer's disease (AD) was originally proposed 20 years ago [1, 2], and during this period it has significantly influenced development of AD-related research. Although it provided a huge amount of data confirming that the accumulation of the amyloid *β*-peptide (A*β*), especially A*β*_1–42_, is directly linked to the development of neurodegeneration, it also to some extent detracted attention from understanding the normal physiological role both of A*β* and its precursor protein, APP. Recently, several attempts have been made to reevaluate the amyloid hypothesis and to suggest new directions in AD research [3–5]. Although our knowledge of the processes involved in A*β* production is rather extensive this has not resulted in any viable therapy despite several promising trials of inhibitors preventing A*β* formation [6]. Moreover, during the last two decades, A*β* toxicity was studied and reexamined in various animal and cellular models suggesting that the toxic A*β* species might be represented by oligomers rather than monomers, fibrils, or plaques [7, 8], and much research has been devoted to the search for pharmacological approaches to prevent A*β* oligomerization as a therapy in AD [9]. 

One of the important concepts developed from the amyloid cascade hypothesis is the realisation that amyloid metabolism is a dynamic process represented by production of A*β* (by *β*- and *γ*-secretases) and its removal from the brain (via perivascular or enzymatic mechanisms) rather than an irreversible pathway of its accumulation leading to cell death and cognitive impairment. As such the enzymes capable of degrading A*β* became a major research and therapeutic target [10–12]. Evaluation of the normal physiological role of A*β* suggests that complete elimination of A*β* from the brain would not be a target in AD therapy since it most likely has a normal physiological role as a regulatory peptide or even as a transcription factor [13–16]. However, by manipulating its levels through improved perivascular drainage or proteolytic degradation might help to prevent accumulation of harmful amyloid species causing cell death and AD pathology [12, 17]. One of the amyloid-degrading enzymes, neprilysin (NEP), has been the main target of our research over many years, and in this paper we will summarize current knowledge of this metallopeptidase and mechanisms to manipulate its activity in disease states.

## 2. General Properties of NEP

Neutral endopeptidase, or neprilysin (NEP), was first described as a neutral proteinase in rat kidney brush border membranes and then purified from rabbit kidney and characterised as a zinc metallopeptidase [18]. Although NEP is abundant in the kidney (about 4% of all membrane proteins), its content in other organs, including the brain, is much lower. NEP was later rediscovered as a brain enzyme responsible for inactivation of the enkephalin family of neuropeptides and given the name enkephalinase [19]. However, it was subsequently shown that NEP is not enkephalin-specific but that it can cleave a wide range of biologically relevant peptide substrates, for example, substance P, and as such it was given the common name, endopeptidase-24.11 [20]. In the literature, NEP is also known as the common acute lymphoblastic leukaemia antigen (CALLA or CD10) since it turned out to be identical with this leukocyte cell surface antigen [21], although to date the substrate(s) and functions of NEP in the immune system have not been identified. NEP was also reported to be identical with a recently described activity termed skin fibroblast elastase which plays a role in skin aging and UVA-induced skin damage [22].

NEP is an oligopeptidase which cleaves peptides containing up to 40–50 amino acids and the most efficiently hydrolyzed substrate is substance P [23]. NEP substrate specificity is rather wide but those for which NEP action has a physiological role in metabolism are rather limited. The principal substrates of NEP *in vivo *appear to be enkephalins, atrial natriuretic peptide, tachykinins, bradykinin, endothelins, adrenomedullin, members of the vasoactive intestinal peptide family, glucagon, thymopentin, and, most significantly in pathophysiological terms, the Alzheimer's disease A*β* peptide.

NEP is a type II integral membrane zinc metalloprotein and does not have a proenzyme form. It is an ectoenzyme with the bulk of its structure, including the active site, facing the extracellular space. Depending on tissue source the *M*_*r*_  of NEP ranges from about 85, 000 to 110, 000 due to differences in its glycosylation [24]. The cDNA cloning of NEP revealed that rat and human enzymes consist of 742 amino acids [25]. The high similarity between human and rodent NEP proteins makes the rat a useful animal model for studying NEP functions and regulation. To date, there are only few characterised endogeneous tissue specific inhibitors of NEP. The first, isolated from bovine spinal cord, was a heptapeptide spinorphin which also inhibited dipeptidyl peptidases and angiotensin-converting enzyme [26]. A decade later, Rougeot and colleagues discovered sialorphin, an exocrine and endocrine signaling mediator, synthesized mostly in the submandibular gland and prostate of rats [27]. The first human NEP inhibitor isolated from saliva was opiorphin which had some pain-suppressive potency [28]. The most potent and widely used NEP inhibitors include phosphoramidon and thiorphan, and the 3D structure of the extracellular domain of NEP in a complex with phosphoramidon has been resolved allowing better understanding of the catalytic properties of the enzyme [29]. One particular feature of the NEP catalytic site is its restricted size which prevents access of large peptides and proteins but allows peptides containing up to 50 amino acid residues. This is consistent with A*β* as a preferred substrate of NEP. Another characteristic feature of NEP is its sensitivity to inhibition by phosphoramidon and thiorphan at nanomolar concentrations. Although a closely related NEP homologue endothelin-converting enzyme (ECE-1) is also inhibited by phosphoramidon, it is only sensitive to micromolar concentrations of the inhibitor and is not affected by thiorphan. 

Despite being originally considered as a unique mammalian membrane endopeptidase, it was subsequently demonstrated that the human genome contains at least seven NEP-like enzymes. This metallopeptidase family is even more abundant in *Drosophila melanogaster* (24 predicted members) and *Caenorhabditis elegans* (22 members), and phosphoramidon-sensitive activities have been identified in these species [30, 31] which makes them useful models for studying functional properties of NEP. In the brain, NEP levels are much lower than in the kidney, and it appears to have mostly neuronal localisation [32] although it was recently reported to be expressed by activated astrocytes [33] and microglia [34]. In peripheral tissues NEP was also found to be transiently expressed on the surface of certain haematopoietic cells and increased NEP levels were found on mature lymphocytes in certain disease states (for review see [35]). It has also been implicated in the progression of a number of cancers, including prostate [36], renal [37], and lung [38] cancer. Another important role of NEP is related to inactivation of the natriuretic peptides *in vivo *and as such NEP inhibitors have been explored as potential cardiovascular and renal therapeutics.

The human *NEP* gene is located on chromosome 3 and exists in a single copy which spans more than 80 kb. It is composed of 24 exons and is highly conserved among mammalian species [39]. Expression of the *NEP* gene is controlled through two distinct promoters [40] whose role differs between cell types, although both promoters show similar characteristics and activity. Three distinct NEP mRNAs have been identified in human and rat which differ only in their 5′-noncoding regions [39, 40]. A gene knockout of *NEP* in mice has been reported in which the animals appeared developmentally normal but the NEP null mice were highly sensitive to endotoxic shock [41]. This observation may reflect a general role of NEP in the metabolism of proinflammatory peptides. NEP knockout mice also showed enhanced aggressive behaviour in the resident-intruder paradigm and altered locomotor activity as assessed in the photobeam system [42]. They also had an increased alcohol and food consumption [43].

## 3. NEP and Neuronal Functions

**Figure 1:**
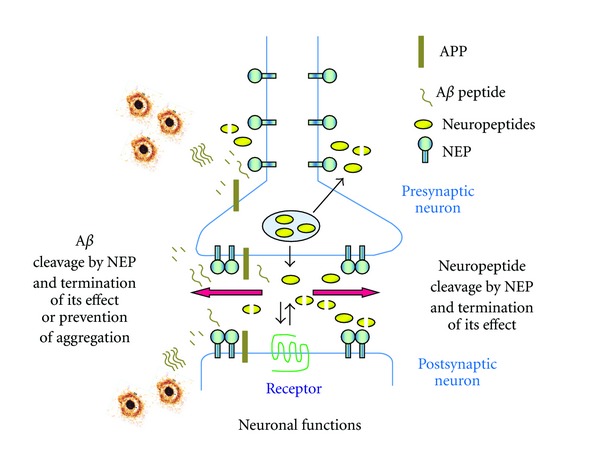
Schematic presentation of NEP localization and functional activity in the brain. NEP being localised pre- and postsynaptically in neuronal cells cleaves its neuropeptide substrates (including A*β*) terminating their properties and as such regulating cellular response to their action and neuronal functions. In the case of A*β*, NEP also prevents accumulation and aggregation of toxic amyloid oligomers. All symbols are explained in the figure.

In the brain, NEP is mainly located on neuronal cells, especially in the striatonigral pathway [44], although it is also present in the hippocampus, where it functions to inactivate somatostatin, and in cortical regions [45]. Pre- and postsynaptic localization of NEP in the nervous system further emphasizes its important role in neuronal function [46] and this is schematically reflected in Figure 1. The enzyme has also been found in Schwann cells in the peripheral nervous system [47]. The significant increase in the expression of NEP by Schwann cells after axonal damage suggests that this enzyme could play a role in axonal regeneration [48]. 

The functional role of NEP in the brain is primarily determined by the physiological properties of its substrates and the roles they play in the nervous system (see Table 1). As such NEP was linked to such brain functions as LTP, synaptic plasticity, motor functions and locomotion, memory, anxiety, pain, hyperalgesia, circadian rhythms, sleep, fatigue, water homeostasis, blood-brain barrier integrity, and neuroinflammation. It plays a certain role in stroke pathology [49], pathophysiology of itch [50], attenuates central functions of baroreceptors [51], food intake, hormonal release, cardiovascular regulation, thermoregulation, stress [52], and anxiolytic response [53]. It also participates in dendrite elongation and the maturation of dendritic spines [54]. NEP was also suggested to play a major role in nociception activating the initial stage of nociceptin metabolism at the spinal cord level [55]. A role for NEP in memory has been confirmed in our experiments with *i.c.* injections of its inhibitors (phosphoramidon and thiorphan) to rats resulting in disruption of memory and neuronal plasticity [56–60]. In addition to these important neuronal functions of NEP, it is also now considered as a major amyloid-degrading enzyme and mechanisms of its regulation and reactivation have been extensively studied in the last decade [12]. Although the precise physiological properties of A*β* peptides are still far from being fully understood, the accumulating evidence suggests that they can act as modulators of neuronal function and synaptic plasticity [61] and the role of NEP in regulating concentrations of A*β* at functional levels can be important for normal brain activity.

## 4. NEP and Amyloid Metabolism

The ability of neprilysin to catabolise *β*-amyloid peptide was first demonstrated *in vitro* by Howell and colleagues [82] and then confirmed *in vivo* [83, 84]. It was demonstrated that NEP knockout mice have increased levels of A*β* peptides in the brain and administration of the neprilysin inhibitor thiorphan to rats led to increased A*β* levels [83, 85]. On the contrary, *NEP* gene transfer to AD transgenic mice was able to reverse amyloid-like pathology and improve animal behaviour [86–88]. Importantly, it was shown that NEP is the most potent A*β*-degrading enzyme in the brain [89] and can degrade not only monomeric forms of A*β* but also its more toxic oligomers [90]. N-terminally truncated forms of A*β* (A*β*_x-42_) and pyroglutamyl modified A*β*_3–42_ are also major contributors to the amyloid pathology of AD due to their abundance in AD brain and their cell toxicity [91]. Although the pyroglutamyl A*β* species have increased resistance to degradation by aminopeptidases [92], the comparative susceptibility of these peptides to NEP activity has not been adequately quantified to date. 

Studies both *in vivo* and *in vitro* have now strongly linked NEP with the pathogenesis of AD and made it a viable therapeutic target. Further *in vivo* studies, including our own work, have indeed demonstrated that NEP mRNA, protein and activity levels decline with age in the cortex and hippocampus of rodents and humans [58–60, 93, 94] and also are reduced in the AD brain [95]. Decreased NEP levels and activity were also reported under such pathological conditions leading to AD, as ischemia or hypoxia [33, 93]. Our studies also demonstrated that prenatal hypoxia leads to reduced NEP protein and activity levels in the cortex and hippocampus of rats during their postnatal life [58–60]. 

Decreased NEP expression in the vasculature was also suggested to be responsible for the development of cerebral amyloid angiopathy found in AD patients [96]. However, along with the age-related and pathology-induced decrease of NEP expression seen in neuronal cells, it was reported that NEP is upregulated in reactive astrocytes surrounding amyloid plaques in AD transgenic mice which could contribute to some compensatory mechanisms [97]. On the contrary, Hickman and colleagues have reported an age-dependent decline of NEP and other amyloid-degrading enzyme expression in microglia resulting in decreased A*β* clearance [34]. Apart from the decline in NEP expression, age-related decrease of NEP capability to degrade A*β* might be due to enzyme oxidation [98] or conformational inactivation, for example, by amyloid peptide [99].

In addition to NEP, its homologue, neprilysin-2 (NEP2), was also characterised in the brain [100]. Although NEP2 is the closest NEP homologue, it has different properties, in particular, in cellular localization. NEP2 has two alternatively spliced forms, one of which is a soluble secreted form, also known as soluble, secreted endopeptidase (SEP) [101]. In the CNS, NEP2 is mainly localized in the cortex and hippocampus and is characteristic to specific neuronal populations [100, 102]. Despite the fact that NEP2 has a broad repertoire of substrates, its physiological role, apart from in male fertility, still is largely unknown. NEP2 was shown to degrade A*β* *in vitro *[89, 103] and recently Hafez and colleagues using gene knockout and transgenic animals have demonstrated that NEP2 contributes to A*β* degradation *in vivo* [104]. Recently it was demonstrated that NEP2 and NEP mRNA expression is altered in the AD-susceptible brain areas of patients with MCI compared to nonimpaired subjects. Moreover, NEP2 enzymatic activity in the mid-temporal and mid-frontal gyri of MCI and AD subjects was lower compared to controls and was associated with the level of cognitive decline [105]. However, at present, mechanisms of NEP2 cell specificity and regulation of its expression and activity have not been sufficiently addressed and further studies are required to estimate the role of this NEP homologue in pathogenesis of AD and to estimate its therapeutic value.

## 5. Modulation of Neprilysin Expression

Reports on age- and AD-related NEP decline have induced an intensive search for means to upregulate NEP gene expression and enzyme activity. *NEP* gene delivery studies have suggested that not only intracerebral injections of *NEP*-bearing constructs can have an antiamyloid effect in AD animal models [87] but that intraperitoneal injections of a lentivirus vector expressing NEP fused with the ApoB transport domain could also reduce A*β* burden and increase synaptic density in the brain of AD transgenic mice [106]. This opened up the development of non-invasive therapeutic approaches for potential treatment in patients with AD. One such approach has utilised a novel system for injection of an NEP coding plasmid into skeletal muscle via a syringe electrode [107]. Injected in this way, hNEP was detected in the muscle, serum, and brain of treated mice even 30 days after injection with minimal damage at the site of electrotransfer. Another, *ex vivo NEP* gene delivery method, was also suggested by Selkoe and colleagues who implanted primary fibroblasts, expressing a secreted form of NEP, into the brain of APP transgenic mice which induced robust clearance of amyloid plaques at the site of engraftment [108]. An interesting approach based on the observation that brain and plasma A*β* are in equilibrium through transport mechanisms [109] was developed by Hersh and colleagues, who found that in AD transgenic mice overexpressing NEP in erythrocytes or leukocytes there was a reduced A*β* burden in the brain [110, 111]. An alternative strategy of expressing a secreted, soluble form of NEP in the plasma through an adenovirus construct was also effective in clearing brain A*β* yet did not affect the plasma levels of other peptide substrates of NEP such as bradykinin or substance P [112]. Expressing NEP in plasma in this way could also provide a simple but effective system to maintain and monitor long-term activity of this amyloid-*β*-degrading peptidase. Along with developing methods of NEP upregulation, the optimal timing of NEP overexpression has also been examined suggesting that earlier upregulation of NEP levels was more beneficial in alleviating symptoms in a mouse model of AD [113].

Apart from targeted gene delivery, strategies for pharmacological NEP regulation have also been intensively studied in the last ten years. Cell culture studies have demonstrated that NEP activity can be increased by, among other compounds, a component of green tea extract, EGCG [114] and other plant extracts and polyphenols (e.g., [115]). Saido and colleagues have suggested that elevated levels of NEP substrates could upregulate NEP by a feedback control mechanism [116]. However, after screening a wide range of NEP neuropeptide substrates, they have found that only somatostatin was capable of upregulating NEP activity in primary neuronal cells. They have also suggested a possible mechanism of NEP activation involving somatostatin receptor subtypes 2 or 4, but these studies have not resulted in any further development of somatostatin receptor agonists for therapeutic application in AD. A 24-residue peptide, humanin, originally isolated from the brain of an AD patient, which has neuroprotective properties and decreases brain A*β* levels in animal models, was shown to mediate its A*β*-lowering effects by increasing NEP expression levels and could also provide a strategy for enhancing amyloid clearance [117]. Another receptor-mediated mechanism for pharmacological upregulation of NEP is the peroxisome proliferator activated receptor-*δ* (PPAR*δ*) whose selective agonist, GW742, was shown to activate the *NEP* promoter driving luciferase expression in transfected HEK293 cells [118].

A completely new direction of studies linking the amyloid cascade hypothesis and NEP to the pathogenesis of AD has emerged from studies of the role of the C-terminal APP intracellular domain (AICD), released by *γ*-secretase activity, in the regulation of NEP transcription [119]. AICD is an approximately 6 kDa peptide which is present as a number of species of which the major form is 50 amino acids long but AICD48 and 51 species are also detectable [120, 121]. It is still unclear whether all of the isoforms of AICD are equally competent in transcriptional regulation. Despite being controversial and disputed by some other authors (e.g., [122–124]), the role of AICD in regulation of *NEP* has been confirmed by demonstrating that AICD binds to the *NEP* promoter in neuronal cells expressing high levels of NEP while in low NEP expressing cells, the *NEP *promoter is repressed by histone deacetylases (HDACs) [125]. This AICD activating effect was shown to be cell specific and even cell age dependent which may explain some of the contradictions in the literature [126–128]. Moreover, it was established that formation of transcriptionally active AICD depends on the particular APP isoform expressed (specifically APP_695_) and requires the active *β*-secretase (amyloidogenic) pathway [126, 129]. Apart from NEP, AICD activates expression of several genes and their number is steadily increasing [130, 131]. An important functional link confirming the role of AICD and gene activation was reported by Xu and colleagues [132] who found that AICD binds the MED12 unit of the mediator RNA polymerase II complex. This finding confirms AICD transcriptional activity [133] and validates other AICD-dependent genes such as aquaporin-1, MICAL2, and fibronectin-1 [132].

The fact that *NEP* gene expression is repressed in neuronal cells via competitive binding of HDACs to its promoter [125] has prompted us to look at the HDAC inhibitors which might reactivate NEP gene expression. As we have found in human neuroblastoma SH-SY5Y cells, trichostatin was able to activate NEP expression at the mRNA and protein levels and also increase its activity. More important from the therapeutic point of view was our observation that a clinically available antiepileptic drug valproic acid (VA) was also able to activate the NEP gene not only in cellular but in animal models as well [59, 125]. Moreover, injections of VA to AD transgenic mice were shown to decrease amyloid-related toxicity and improve animal behaviour although the authors had not considered to analyse levels of NEP expression and activity in their paradigm [134]. Our own animal studies have further demonstrated that administration of VA to rats with reduced levels of NEP expression in the brain due to prenatal hypoxia resulted in increased NEP activity in the cortex and hippocampus and improvement of animal short-term memory [58] which can be linked with the role of NEP in dendritic spine formation and restoration of neuronal circuits [59, 60]. The role of histone modifications in downregulation of the *NEP* promoter under hypoxic conditions has also been demonstrated by Wang and colleagues in primary cortical neuronal cells who demonstrated that NEP mRNA levels could be restored by VA administration to cells prior to hypoxia [72]. These studies revise the role of such a widely used antiepileptic drug as VA in regulation of neuronal gene expression and its protective role in neurodegeneration [135]. However, they also underlie the necessity for design of more specific HDAC inhibitors for targeted activation of NEP or other neuronal and, specifically, AD-related genes. Indeed, a recent report specified that inhibitors of class 1 HDACs reverse contextual memory deficits in an AD-mouse model [136]. This opens an avenue for retrospective analysis of the effect of VA or other HDAC inhibitors on development of AD. 

Another therapeutically approved compound which was shown to modulate NEP expression via AICD-dependent mechanisms is the tyrosine kinase inhibitor, Gleevec (imatinib, STI-571), which was shown to elevate AICD levels and increase NEP mRNA and protein levels [137]. Although other authors failed to support this observation [138], recent work by Bauer and colleagues clearly demonstrated that the imatinib- (Gleevec-) induced NEP increase is APP and AICD dependent [127].

Importantly, in prostate cancer, NEP expression is downregulated by extensive hypermethylation of the promoter region and reexpression of neprilysin by treating the animals with the demethylating agent 5-aza-2′-deoxycytidine was able to inhibit tumor formation in the prostates of athymic mice [139, 140]. According to our data, downregulation of NEP in neuronal cells is not due to hypermethylation of its promoter and cannot be reactivated by 5-aza-2′-deoxycytidine which confirms cell specificity of *NEP* gene regulation [125].

**Figure 2:**
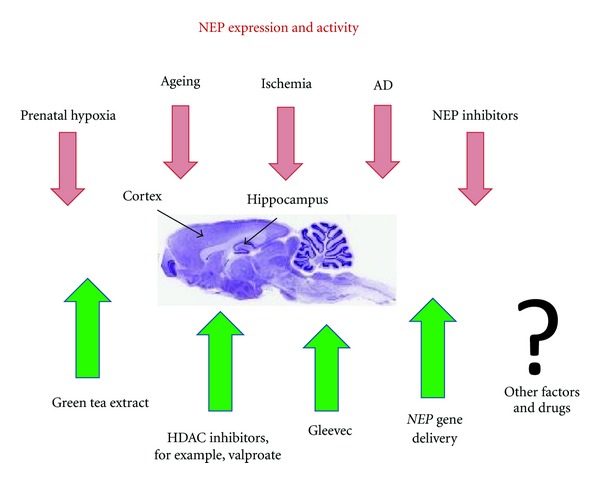
Effects of various experimental conditions on NEP activity *in vivo*. As explained in the text, NEP expression and activity in brain cortex and hippocampus (the structures which are characterised by accumulation of amyloid deposits) decreases with age and is also decreased after prenatal hypoxia, ischemia, or in the case of AD. In animal models, NEP activity can be modulated by its inhibitors affecting such brain functions as learning and memory. Mechanisms which can control and upregulate NEP expression and increase its activity include targeted NEP gene delivery, regulation of its promoter via inhibition of HDACs or pharmacologically by green tea extract (or EGCG) or Gleevec.

As mentioned above, green tea extracts EFLA85942 and EGCG increase NEP expression and activity in human neuroblastoma SH-SY5Y, SK-N-SH, and NB7 cells ([114] and our own unpublished data). Extending these studies to animal models, we have found that prolonged EGCG administration to rats via osmotic minipumps was able to increase NEP activity in hypoxic rats to the levels recorded in control age-matched animals. Moreover, administration of EGCG has also improved performance of animals in the radial maze and improvement of short-term and long-term memory in the novel object recognition test [57]. This further supports the role of NEP in memory and extends the list of biologically active compounds which might be beneficial for prevention of cognitive deficit characteristic to AD pathology (Figure 2).

## 6. Concluding Remarks

Twenty years on from the formulation of the amyloid cascade hypothesis, there have been no successful clinical trials in AD. Several reasons for this can be suggested, for example, initiation of trials in patients in which neuronal loss and damage is already too far advanced, emphasizing the need for early diagnosis and good biomarkers. Also, late onset disease may well reflect defects in clearance mechanisms for A*β* rather than in the enhanced synthesis which occurs in early onset cases [141]. Hence, strategies to promote clearance, such as elevation of NEP expression and activity, may represent new opportunities for therapeutic intervention, either alone or in combination with other strategies. As follows from the detailed analysis of NEP properties and function, this enzyme plays an important role in brain function and disruption of its natural metabolic roles leads to various pathological conditions both centrally and in the periphery. Upregulation of NEP expression in such diseases as AD or prostate cancer has already been shown to be beneficial in animal models and various approaches have now been developed to activate this enzyme in cells and organisms. The discovery of epigenetic and pharmacological mechanisms for controlling NEP activity suggests a possibility for design of a preventive therapeutic strategy in AD and other age-related human diseases. Taking into account the wide substrate repertoire of NEP, there might be a cohort of functions which can be maintained by NEP modulators such as learning and memory, pain and inflammation, depression and anxiety, and further research of the precise molecular mechanisms involved in tissue and cell-specific regulation of this peptidase might give us a powerful tool to improve human health and wellbeing.

## Figures and Tables

**Table 1:** Functional role of NEP and some of its substrates in the CNS.

NEP substrates	Functions
Adrenomedullin	Vasodilator; tolerance to oxidative stress and hypoxia; inhibition of dendrite formation in the cerebral cortex [62], anxiety, pain [63]
Amyloid *β*-peptide	LTP, synaptic plasticity, memory, AD pathology [64]
Angiotensin I	Precursor to angiotensin II; enhances baroreceptor sensitivity [51]
Angiotensin II	Central cardiovascular regulation; attenuates baroreceptor sensitivity [51]
Bradykinin	Vasodilator; pain, hyperalgesia [65]; regulation of astrocyte calcium levels [66]
Cholecystokinin-8	Feeding behaviour, satiety, anxiety, obesity [67]
Corticotropin	Sleep, fatigue [68]
Dynorphins	Learning and memory, emotional control, stress response, pain [69]
Endomorphin	Pain, analgesic effect [70]
Enkephalins	Pain perception, cognitive functions, affective behaviours, locomotion [71]
Endothelin-1	Vasoconstriction, effects on water homeostasis and blood-brain barrier integrity, neuroinflammation, stroke [72]
Gastrin	Circadian rhythms [73], pathophysiology of itch [50]
Neuropeptide Y	Food intake, hormonal release, circadian rhythms, cardiovascular regulation, thermoregulation, stress response, anxiety and sleep [52]
Neurotensin	Modulation of dopamine signalling; dendrite elongation and the maturation of dendritic spines [54]
Oxytocin	Sexual arousal, bonding, stress, anxiolytic response [53]
Somatostatin	Motor activity, sleep, sensory processes, cognitive functions [74]
Substance P	Pain and inflammation [75], drug addiction [76], learning and memory [77], depression and anxiety [78, 79], itching [80]
VIP	Circadian rhythm [81]

## Abbreviations

A*β*: Amyloid *β*-peptide
AD: Alzheimer's disease
ADE: Amyloid-degrading enzyme
AICD: APP intracellular domain
APP: Amyloid precursor protein
EGCG: Epigallocatechin gallate
HDAC: Histone deacetylase
IDE: Insulin-degrading enzyme
MED: Mediator
NEP: Neprilysin
PKC: Protein kinase C
PPAR: Peroxisome proliferator-activated receptor
SNP: Single nucleotide polymorphism
VA: Valproic acid.
